# Retraction: *Rhodomyrtus tomentosa* as a new anticancer molecular strategy in breast histology via Her2, IL33, EGFR, and MUC1

**DOI:** 10.3389/fphar.2026.1791496

**Published:** 2026-01-29

**Authors:** Frontiers Editorial Office

The Journal retracts the 2024 article cited above.

Following publication, concerns were raised regarding the integrity of the images in the published figures on PubPeer, where several partial duplications across **Figures 5–8** are documented.

The authors failed to provide a satisfactory explanation during the investigation, which was conducted in accordance with Frontiers’ policies. As a result, the data and conclusions of the article have been deemed unreliable, and the article has been retracted.

This retraction was approved by the Chief Executive Editor of Frontiers. The authors do not agree to this retraction.

Frontiers would like to thank Sholto David for contacting the journal regarding the published article.

